# MicroRNA-19a contributes to the epigenetic regulation of tissue factor in diabetes

**DOI:** 10.1186/s12933-018-0678-z

**Published:** 2018-02-24

**Authors:** Marco Witkowski, Termeh Tabaraie, Daniel Steffens, Julian Friebel, Andrea Dörner, Carsten Skurk, Mario Witkowski, Bernd Stratmann, Diethelm Tschoepe, Ulf Landmesser, Ursula Rauch

**Affiliations:** 1 Charité Centrum 11, Depart. of Cardiology, Campus Benjamin Franklin, Charité University Medicine Berlin, Hindenburgdamm 30, 12200 Berlin, Germany; 2 Institute of Microbiology and Infection Immunology, Charité University Medicine Berlin, Berlin, Germany; 3 Heart and Diabetes Center NRW, Ruhr University of Bochum, Bad Oeynhausen, Germany

**Keywords:** Diabetes mellitus, Tissue factor, MicroRNA 19a, Coagulation, Vascular inflammation

## Abstract

**Background:**

Diabetes mellitus is characterized by chronic vascular disorder and presents a main risk factor for cardiovascular mortality. In particular, hyperglycaemia and inflammatory cytokines induce vascular circulating tissue factor (TF) that promotes pro-thrombotic conditions in diabetes. It has recently become evident that alterations of the post-transcriptional regulation of TF via specific microRNA(miR)s, such as miR-126, contribute to the pathogenesis of diabetes and its complications. The endothelial miR-19a is involved in vascular homeostasis and atheroprotection. However, its role in diabetes-related thrombogenicity is unknown. Understanding miR-networks regulating procoagulability in diabetes may help to develop new treatment options preventing vascular complications.

**Methods and results:**

Plasma of 44 patients with known diabetes was assessed for the expression of miR-19a, TF protein, TF activity, and markers for vascular inflammation. High miR-19a expression was associated with reduced TF protein, TF-mediated procoagulability, and vascular inflammation based on expression of vascular adhesion molecule-1 and leukocyte count. We found plasma expression of miR-19a to strongly correlate with miR-126. miR-19a reduced the TF expression on mRNA and protein level in human microvascular endothelial cells (HMEC) as well as TF activity in human monocytes (THP-1), while anti-miR-19a increased the TF expression. Interestingly, miR-19a induced VCAM expression in HMEC. However, miR-19a and miR-126 co-transfection reduced total endothelial VCAM expression and exhibited additive inhibition of a luciferase reporter construct containing the *F3* 3′UTR.

**Conclusions:**

While both miRs have differential functions on endothelial VCAM expression, miR-19a and miR-126 cooperate to exhibit anti-thrombotic properties via regulating vascular TF expression. Modulating the post-transcriptional control of TF in diabetes may provide a future anti-thrombotic and anti-inflammatory therapy.

**Electronic supplementary material:**

The online version of this article (10.1186/s12933-018-0678-z) contains supplementary material, which is available to authorized users.

## Background

Diabetes is characterized by a chronic inflammatory state of the vasculature leading to increased cardiovascular complications and death [1]. Endothelial dysfunction sustains a procoagulant state resulting in thromboembolic events in those patients [2, 3].

Being the receptor for FVIIa, tissue factor (TF) is the primary initiator of the coagulation cascade with a crucial role in haemostasis [4]. When in contact with coagulation factors, TF promotes FXa generation and thrombin-induced clotting. Under normal conditions, TF is not present in the blood, its expression in the vasculature, however, strongly increases upon presence of pro-inflammatory cytokines, such as tumor necrosis factor(TNF)α, or advanced glycolysation end products [5–7]. The full length (fl)TF protein is much more thrombogenic compared to the alternatively-spliced (as)TF, promoting angiogenesis and cell survival [8, 9]. Blood-borne TF is derived from vascular wall cells and monocytes and promotes coagulation and vascular inflammation [4, 10–13]. Notably in diabetes with poor glycaemic control, circulating TF in the blood accounts for heightened coagulability and diabetic complications [14, 15].

Recently, the small non-coding microRNA(miR)s have been implicated in diabetes and its cardiovascular complications [16, 17]. Distinct miR expression patterns appear to predict the onset of the disease and may help stratify the risk for thromboembolic events [18]. However, mechanistic insights on how those miRs impact diabetic vascular complications are lacking.

We and others have shown that miRs control TF expression on the post-transcriptional level [4, 19, 20, 22]. It can be assumed that specific miR signatures in the healthy vasculature contribute to the control of blood-borne TF expression and alterations in this regulatory network may promote thromboembolic events. In line, we found that the endothelial-derived miR-126, that is reduced under diabetic conditions [21], prevents TF-dependent procoagulability in diabetes [22]. In addition, miR-19a, a member of the miR-17-92 cluster, was shown to impact post-transcriptional TF regulation in cancer cells in vitro [20]. However, beyond its role as an onco-miR, the high expression of miR-19a in endothelial cells (ECs) suggests a central role in endothelial homeostasis. Indeed, miR-19a is involved in vascular functions, including angiogenesis [23], EC apoptosis [24] or atheroprotection via flow-regulated control of endothelial proliferation [25]. On one hand, reduced levels of circulating miR-19a in patients with diabetes-related complications, such as ischemic stroke, point out a potential role in the pathogenesis of thromboembolism [26]. On the other hand, miR-19a regulates glycogen synthesis and is down regulated in leptin receptor-deficient mice suggesting a protective role in diabetes [27]. Whether vascular expression of miR-19a reduces procoagulability in diabetes has not been studied yet.

In the present study, we sought to investigate the role of circulating miR-19a in the post-transcriptional control of vascular TF expression in poorly controlled diabetes.

## Research design and methods

### Patient study

The study protocol was approved by the local ethics committee and was performed in accordance to the ethics principles in the Declaration of Helsinki. Prior to participation in the study each patient gave a written informed consent. 44 patients with known diabetes mellitus type 2 (hereinafter referred to as diabetes) admitted for poor glycaemic control at the Heart and Diabetes Center NRW Bad Oeynhausen, Germany, were included in the study [28]. To assess the impact of miR-19a expression, the patients were divided into two groups according to their miR-19a expression (lower than the median of miR-19a expression, n = 22; higher than the median of miR-19a expression, n = 22). Table 1 describes the patient characteristics. Peripheral blood was obtained by venepuncture into heparin, citrate or EDTA tubes upon admission. miR-19a expression was measured via TaqMan PCR in citrated blood plasma. Protein amounts for TF, VCAM-1, Intercellular adhesion molecule (ICAM)-1, endothelin, and E-selectin in citrated plasma were assessed using a specific ELISA system. TF activity in plasma was analysed by a factor Xa chromogenic assay.

**Table 1:** Patient characteristics

Characteristics	Low miR-19a	High miR-19a	p value
miR-19a	0.620 ± 0.07	4.537 ± 0.98	< 0.0001
miR-126	2.386 ± 0.31	13.26 ± 2.80	< 0.0001
Sex (m/f)	15/7	18/4	n.s.
Age (years)	67 ± 1.7	62 ± 1.9	< 0.05
History of stroke (%)	13.6	9.0	n.s.
History of MI (%)	18.1	4.5	n.s.
CAD (%)	50.0	31.8	n.s.
Hypertension (%)	100	100	n.s.
PAD (%)	27.2	22.7	n.s.
Diabetic neuropathy (%)	81.8	77.2	n.s.
Diabetes years	15 ± 2.3	11 ± 2.1	n.s.
BMI (kg/m^2^)	30.2 ± 1.3	34.2 ± 1.7	n.s.
CRP (mg/dL)	0.83 ± 0.2	1.0 ± 0.5	n.s.
HbA1c (%)	7.7 ± 0.2	8.7 ± 0.3	n.s.
HbA1c (mmol/mol)	61.1 ± 3.1	72.3 ± 3.9	n.s.
Fasting blood glucose (mg/dL)	144 ± 12	146 ± 6	n.s.
ACE-inhibitor (%)	40.9	50.0	n.s.
AT II antagonist (%)	31.8	31.8	n.s.
Diuretics (%)	54.5	72.7	n.s.
Statins (%)	54.5	50.0	n.s.
Anti-diabetic drugs: insulin (%)	72.7	68.1	n.s.
Metformin (%)	36.3	68.1	< 0.05
Sulfonylureas (%)	27.2	4.5	< 0.05
Acarbose (%)	13.0	9.0	n.s.
Glinides (%)	4.5	13.6	n.s.

*ACE* angiotensin-converting enzyme, *AT II* angiotensin II, *BMI* body mass index, *CAD* coronary artery disease, *CRP* c-reactive protein, *HbA1c* glycated haemoglobin, *MI* myocardial infarction, *PAD* peripheral artery disease

### ELISA experiments

The TF plasma concentrations as well as levels of VCAM-1, ICAM-1, endothelin, and e-selectin were assessed by using a specific ELISA system from American Diagnostica according to manufactures instructions [29].

### TF activity

The measurement of TF activity was performed as described before using a self-designed assay [30–32]. To determine the TF activity 20 µL citrate plasma was added to 160 µL of a solution containing 2 nM FVIIa, 150 nM factor X, and 5 mmol/L CaCl_2_. The generation of FXa was stopped after 30 min by adding EDTA buffer (50 mmol/L Bicine, pH 8.5, 100 mmol/L NaCl, 25 mmol/L EDTA, 1 mg/mL BSA). Then spectrozyme (0.5 mmol/L final concentration), the chromogenic substrate of FXa, was added to each sample. The optical density was determined at 405 nm by using an ELISA plate reader at 37 °C (Molecular Devices). TF activity units were assessed by a standard curve. The standard curve is constructed by plotting the mean slope absorbance value measured for each lipidated TF standard against its corresponding concentration [pg/mL]. The activity (generation of Factor Xa) exhibited by 1 pg of lipidated TF corresponds to 1 arbitrary TF-activity unit. The recombinant FVIIa (NovoSeven) was kindly provided by Novo Nordisk.

### Cell culture

HMEC ordered from ATCC were maintained in MCBD 131 medium (Gibco) + 10% FBS (Gibco) + 100 U/mL penicillin/streptomycin (PAA) + 2 mM l-Glutamin (PAA) + 0.05 mg/mL Hydrocortison. HMEC were used for experiments until the 15th passage. Human embryonic kidney (HEK) cells were cultured in DMEM + 10% FBS + 100U/mL penicillin/streptomycin.

THP-1 cells were grown in RPMI 1640 medium (Gibco) + 10% FBS + 1% penicillin/streptomycin.

### Transfection and stimulation experiments

HMEC cells were transfected with 200 nM negative control mimic (miRIDIAN micro RNA, Dharmacon), an inhibitor negative control (miRIDIAN micro RNA, Dharmacon), 200 nM miR-19a mimic (hsa-miR-19a-3p, HMI0344, MISSION miRNA mimic, Sigma), 200 nM anti-miR-19a (hsa-miR-19a-3p inhibitor, HSTUD0343, MISSION, Sigma) or a miR-126 mimic (has-miR-126-3p, MISSION miRNA mimic, Sigma) using the siRNA transfection reagent interferin (VWR) according to manufacturer’s protocol. 24 h post transfection cells were starved in MCBD 131 medium (Gibco) for 2 h and then stimulated with 10 ng/mL TNF-α for 2 h for gene expression analysis and 6 h for protein expression of the TF splice variants. THP-1 cells were stimulated with LPS (10 µg/mL) and miR expression and TF activity was assessed.

### Dual luciferase reporter assay

To perform the dual luciferase reporter assay, HEK were co-transfected with 200 nM control miR, miR-19a or miR-126 mimic and a luciferase reporter vector, miTargetTM 3′UTR target clone pEZX-MT01 harbouring the *F3*-3′UTR (GeneCopoeia) using interferin (VWR). The reporter plasmid expresses renilla luciferase as a house keeping control and firefly luciferase combined with the TF-3′UTR as a reporter for the miR-mediated repression of the TF mRNA. 24 h post transfection the luciferase assay was performed using the dual luciferase reporter system (Promega) according to manufacturer’s protocol and the ratio firefly/renilla luciferase activity was measured using a luminometer.

### Real-time PCR and western blot analysis

For real-time PCR, total mRNA was isolated with peqGOLD Trifast (Peqlab) for cell culture experiments or using the mirVana Paris Kit (Life Technologies) for patient blood plasma. Gene expression was determined using self-designed FAM-tagged TaqMan^®^ gene expression assays (life Technologies) for flTF and asTF (for details see [33]). The expression of miR-19a and miR-126 was analysed with a FAM tagged TaqMan^®^ gene expression assay (hsa-miR-19a-3p—000395 and hsa-miR-126-3p—002228). Relative gene expression was determined via the comparative C(t) (ΔΔCt) method with Glyceraldehyde 3-phosphate dehydrogenase (GAPDH)- Hs99999905_m1 as endogenous control for mRNA and U6 snRNA—001973 for miR as endogenous control. Western blots were performed as described before [11]. Antibodies were used for flTF (4 µg/mL, 4501, Sekisui Diagnostics), asTF (1 µg/mL, self-generated), VCAM-1 (concentration 0.6 µg/mL, E1E8X, cell signalling) as well as GAPDH (0.6 µg/mL, CB1001, Calbiochem).

### Statistical analysis

The statistical analyses have been performed using the commercially available software GraphPad Prism 5. In the patient cohort a Mann–Whitney U test has been performed for pairwise comparisons between two independent groups. Spearman’s correlation coefficient analysis was used to assess associations between several parameters of the cohort. In cell culture experiments differences between two groups were examined using Student’s t test (2-tailed). For comparisons of 1 parameter between more than two groups a 1-way ANOVA with the Turkey’s post hoc test was performed. Data are represented as mean ± SEM. p values < 0.05 were considered statistically significant.

## Results

### Plasma miR-19a expression correlates with reduced TF-related thrombogenicity in patients with diabetes

**Fig. 1:**
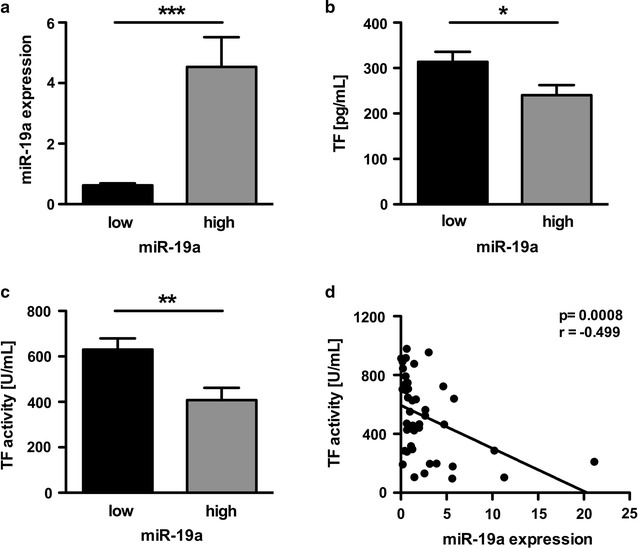
Plasma miR-19a correlates with reduced TF protein and TF-mediated thrombogenicity in patients with diabetes. **a** Expression of miR-19a in both groups with either low or high miR-19a expression. Differences in **b** TF protein and **c** TF activity in the plasma depending on low or high miR-19a plasma expression. **d** Correlation between miR-19a and TF activity. Data are expressed as mean ± SEM. n = 44, *p < 0.05, **p < 0.005, ***p<0.0001

To assess a potential role of miR-19a in diabetes related thrombogenicity we conducted a study enrolling 44 individuals with known diabetes admitted for insufficient glycaemic control (mean HbA1c for all patients was 8.2 ± 0.23% or 66.7 ± 2.64 mmol/mol). In all patients, we observed a broad range of miR-19a expression (from 0.03 to > 20 miR-19a expression normalized to U6 snRNA). According to the plasma miR-19a expression, the patients were divided into two groups with either high miR-19a expression (n = 22) above the median (1.258 miR-19a expression level) or low miR-19a expression below the median (n = 22) (Table 1). Quantitative RT-PCR revealed a significant difference in miR-19a expression in both groups (Fig. 1a). Except for age, there were no differences in the two groups regarding body mass index, diabetes duration, fasting blood glucose, or HbA1c. However, in the group with high miR-19a expression the patients were younger and a higher percentage of treatment with metformin and a lower percentage of sulfonylurea treatment was seen. Interestingly, the patients with low miR-19a expression showed more thromboembolic events, which was however not statistically significant in this cohort (Table 1).

The patients with a high miR-19a expression showed significantly lower protein levels of TF in the blood as compared to patients with a low miR-19a expression (Fig. 1b). Accordingly, TF activity assessed as FXa generation was significantly decreased in patients with high miR-19a levels as compared to the group with low miR-19a levels (Fig. 1c). In line with these observations, miR-19a plasma levels showed a significant negative correlation with the TF protein (not shown) and TF activity in all patients (Fig. 1 d).

### Endothelial miR-19a correlates with miR-126: implication in vascular inflammation

**Fig. 2:**
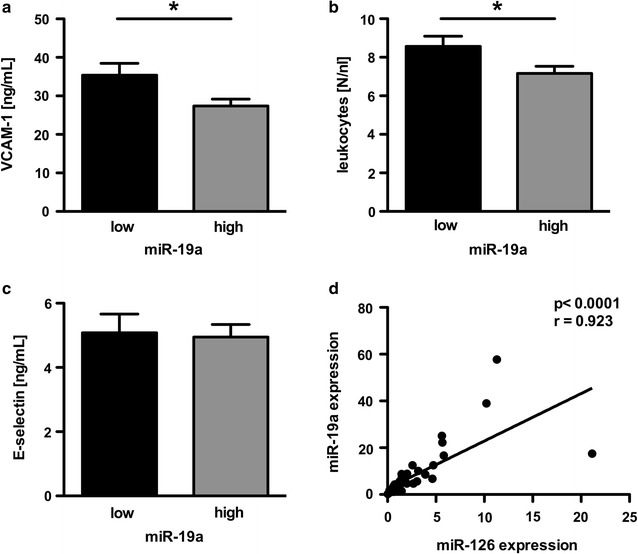
miR-19a is associated with reduced vascular inflammation and correlates with miR-126 in patients with diabetes. Relation between the expressions of **a** the inflammatory protein VCAM-1, **b** leukocytes, and **c** E-selectin in patients with either low or high miR-19a expression. **d** Positive correlation of miR-19a and miR-126 in the patients with diabetes. Data are expressed as mean ± SEM. n = 44, for A and C n = 36, *p < 0.05

To investigate the role of miR-19a in vascular inflammation as part of the pathogenesis of diabetes, pro-inflammatory factors were assessed. miR-19a expression was associated with a reduced grade of vascular inflammation. In the group of patients with high miR-19a expression we observed a lower protein amount of VCAM-1 but not endothelin (data not shown) or E-selectin (Fig. 2a, c). High miR-19a expression was associated with a lower leucocyte count than in the group with low miR-19a expression (Fig. 2b). In the group with enriched plasma miR-19a levels we also observed an increased expression of miR-126 (Table 1). Both miRs showed a significant correlation in the plasma of patients with diabetes (Fig. 2d).

### miR-19a down regulates asTF and flTF in HMEC-1 cells

**Fig. 3:**
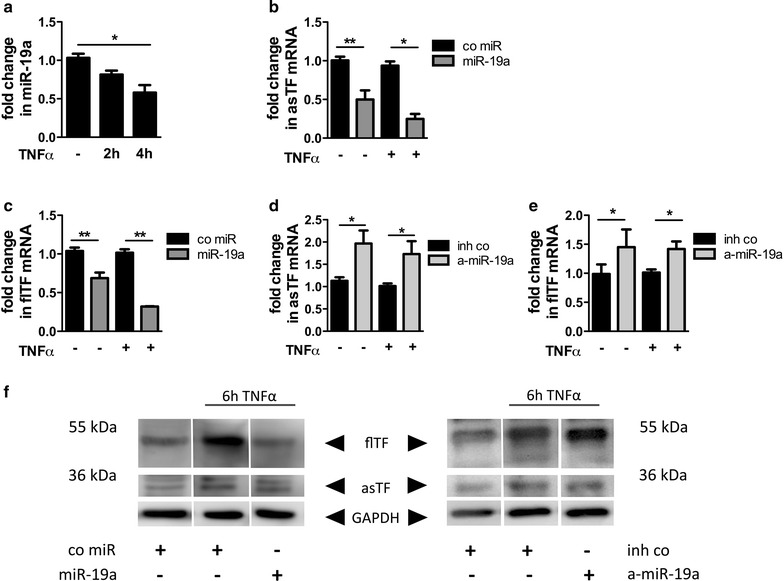
miR-19a reduces the expression of both asTF and flTF in ECs. miR-19a expression in HMEC stimulated with 10 ng/mL TNF-α for 2 or 4 h (**a**). HMEC were cultured for 24 h and then transfected with a control mimic or miR-19a as well as an inhibitor control or anti-miR-19a. 24 h post transfection the cells were left untreated or stimulated with 10 ng/mL TNF-α. **b**, **d** asTF and **c**, **e** flTF mRNA under basal conditions and 2 h after stimulation with TNF-α. **f** Representative western blot shows the protein expression of flTF and asTF upon transfection with miR-19a or anti-miR-19a and their specific controls 6 h post TNF-α stimulation. Data are represented as mean ± SEM. *p < 0.05, **p < 0.01, n ≥ 5

To further assess the miR-mediated regulation of TF expression within the endothelium, HMEC were cultivated. ECs do not express considerable amounts of TF under basal condition, while TNF-α strongly induces endothelial TF expression. In the following experiments, TNF-α served as an inducer for TF expression under inflammatory conditions. We found a high expression of miR-19a in HMEC that was reduced upon stimulation with TNF-α (Fig. 3a).

To assess the impact of miR-19a on both TF isoforms, HMEC were transfected with a control mimic or miR-19a and the cells were then left untreated or stimulated with TNF-α. Transfection efficiency was confirmed via real-time PCR. The mRNA of asTF and flTF was significantly reduced in HMEC transfected with miR-19a compared to cells transfected with the control mimic under basal conditions and 2 h post stimulation with TNF-α (Fig. 3b, c). In Line, transfection of a specific inhibitor of miR-19a (anti-miR-19a) led to an increase of both asTF and flTF mRNA (Fig. 3d, e). Due to the low TF protein amounts under basal conditions, western blot analysis was performed in HMEC under inflammatory conditions. Upon TNF-α treatment, flTF protein was increased, whereas asTF was only mildly induced. Transfection of miR-19a exhibited a reduction in flTF and asTF protein compared to a control mimic after 6 h of stimulation with TNF-α (Fig. 3f and Additional file 1: Figure S1A, B with densitometric analysis). Accordingly, anti-miR-19a caused an increase in flTF and asTF protein (Fig. 3f and Additional file 1: Figure S1C, D).

### miR-19a controls TF procoagulant activity in THP-1 cells

**Fig. 4:**
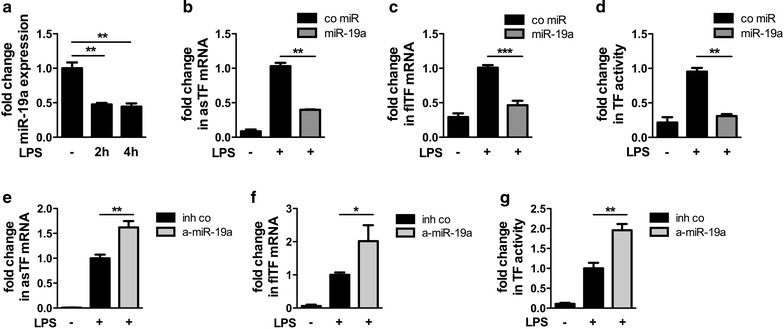
miR-19a reduces TF procoagulant activity in human monocytic cells miR-19a expression in THP-1 cells stimulated with 10 μg/mL LPS for 2 or 4 h (**a**). THP-1 cells were transfected with either a control mimic or miR-19 as well as an inhibitor control or anti-miR-19a. 24 h post transfection cells were stimulated with 10 μg/mL LPS and TF mRNA and TF activity assessed by real-time PCR and a FXa chromogenic assay, respectively. **b**, **e** asTF and **c**, **f** flTF mRNA 2 h following stimulation with LPS. **d**, **g** TF activity 6 h post stimulation with LPS. Data are represented as mean ± SEM. *p < 0.05, **p < 0.01, ***p < 0.001, n ≥ 4

In addition to the vessel wall, monocytes are considered the most important source of TF activity in the blood. The human monocytic cell line THP-1 robustly expressed miR-19a. Treatment of the cells with LPS led to a reduction of miR-19a (Fig. 4a). To study the impact of miR-19a on monocyte TF expression, THP-1 cells were cultivated and transfected with miR-19a or anti-miR-19a. The transfection efficiency was confirmed by Taqman PCR. Next, the cells were stimulated with LPS for 2 or 6 h and TF mRNA and procoagulant activity assessed using Taqman PCR or a FX chromogenic assay, respectively. Transfection of miR-19a exhibited a strong decrease in asTF and flTF mRNA (Fig. 4b, c). Moreover, miR-19a reduced the TF procoagulant activity as compared to a control mimic after LPS stimulation (Fig. 4d). In contrast, anti-miR-19a increased asTF mRNA, flTF mRNA, and the FXa generation under the same conditions (Fig. 4e–g).

### miR-19a and miR-126 in concert impact endothelial VCAM1 expression

**Fig. 5:**
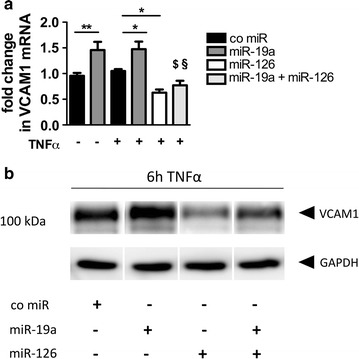
miR-19a induces endothelial VCAM1 expression, which is counter acted by miR-126. HMEC were cultured overnight and then transfected with a control mimic, miR-19, miR-126, or both miR-19a and miR-126 together. 24 h post transfection the cells were left untreated or stimulated with 10 ng/mL TNF-α. **a** VCAM1 mRNA expression analysed by real-time PCR. **b** Representative western blot shows the protein expression of VCAM1 6 h post TNF-α stimulation. Data are represented as mean ± SEM. *p < 0.05, **p < 0.01, $ p < 0.0001 vs. miR-19a TNF-α, § n.s. vs. co miR TNF-α, n ≥ 4

To analyse the miR-mediated impact on cell adhesion molecules, HMEC were transfected with miR-19a. The cells were then left untreated or VCAM1 was induced via TNF-α. Interestingly, miR-19a transfection resulted in an increase in VCAM1 mRNA under basal conditions and 2 h post treatment with TNF-α (Fig. 5a). Since our patient data revealed a strong correlation of miR-19a and miR-126, we investigated VCAM expression in the presence of both miRs under inflammatory conditions. In contrast to miR-19a, miR-126 led to a reduction in VCAM mRNA. Importantly, when miR-19a and miR-126 were co transfected, no increase in VCAM1 mRNA could be seen (Fig. 5a).

Since under basal conditions no VCAM1 protein was detectable, the western blot protein measurements were performed under inflammatory conditions. Following treatment with TNF-α for 6 h we observed an increase in VCAM1 upon miR-19a transfection while miR-126 reduced VCAM1 protein. When both miRs were co transfected an overall reduction in VCAM1 protein was observed (Fig. 5b and Additional file 1: Figure S1E).

### Both miR-19a and miR-126 exhibit concomitant inhibition of the *F3* 3′UTR

**Fig. 6:**
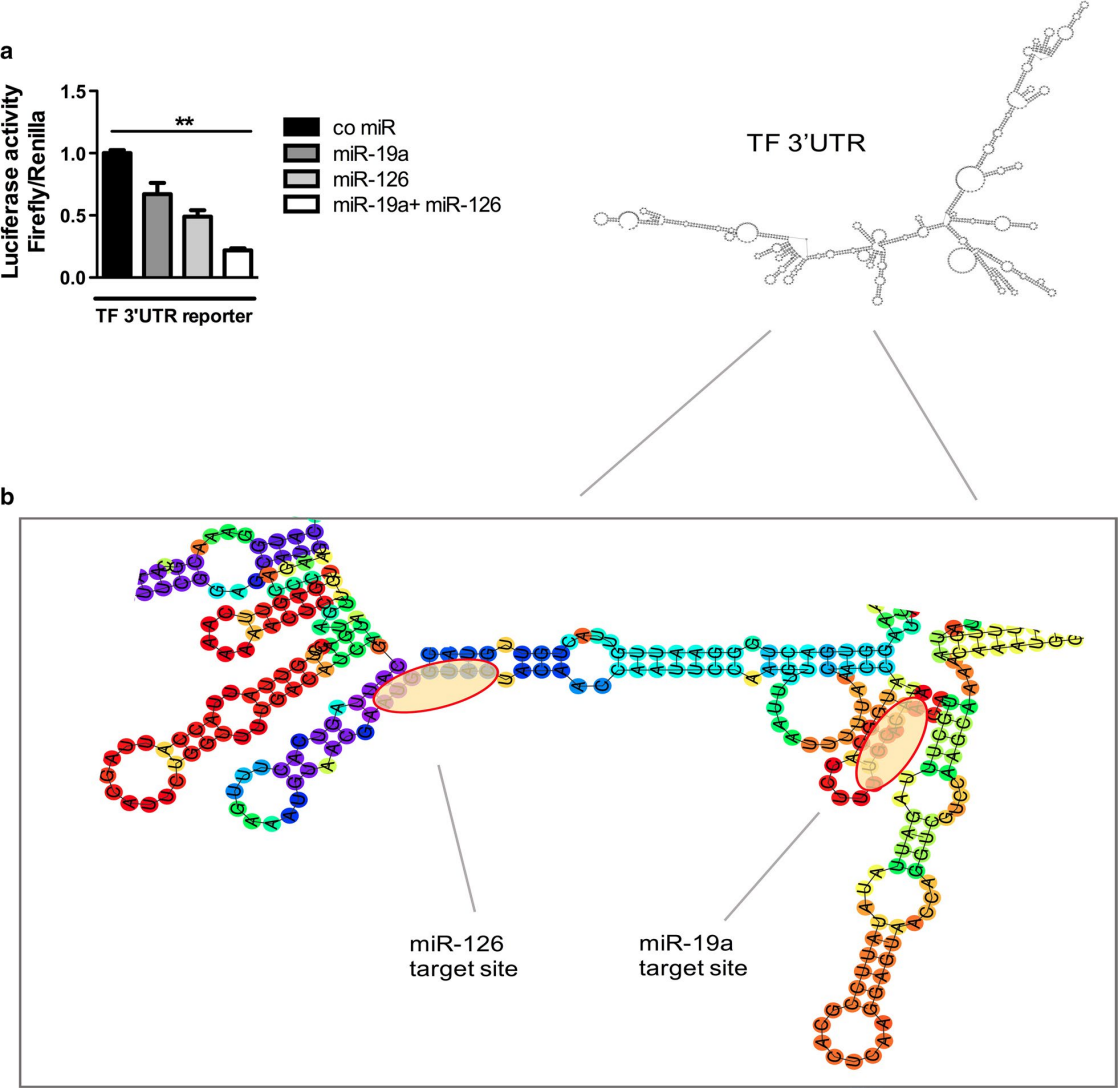
miR-19a and miR-126 cooperatively supress the TF 3′UTR. Hek cells were co transfected with a TF-3′UTR-harbouring reporter plasmid and a control miR, miR-19a, miR-126, or miR-19a and miR-126 together. After 24 h (**a**) the firefly luciferase activity was measured and normalized to renilla luciferase activity. **b** Illustration of the predicted binding sites for miR-19a and miR-126 within the 3′UTR of the TF transcript using the online software RNAfold (http://rna.tbi.univie.ac.at/cgi-bin/RNAWebSuite/RNAfold.cgi). Data are represented as mean ± SEM. **p < 0.01, n ≥ 4

To analyse whether miR-19a and miR-126, which we found to be co expressed in the patients with diabetes, exhibit a cumulative effect on the 3′untranslated region (UTR) of the TF (*F3*) transcript, a luciferase assay was performed in HEK cells. Transfection of miR-19a or miR-126 together with a luciferase-reporter construct containing the *F3*-3′UTR caused a reduction in luciferase activity (Fig. 6a) confirming the miR-depending repression of the TF mRNA. When both, miR-19a and miR-126, were co transfected, a further reduction in luciferase activity was observed.

## Discussion

In this study, we showed that plasma miR-19a expression correlates with reduced TF protein expression and activity in patients with diabetes. High miR-19a levels were associated with reduced vascular inflammation assessed by VCAM-1 protein expression and leukocyte count. A strong correlation was observed between miR-19a and the TF-inhibitory miR-126. We found miR-19a to reduce the TF expression on mRNA and protein level in human ECs as well as TF activity in THP-1. Our data were confirmed by use of anti-miR-19a. While both miRs had a differential effect on VCAM1 expression, miR-19a and miR-126 cooperatively inhibited the *F3*-3′UTR in vitro.

### miR-19a targets the TF 3′UTR: Reduced procoagulability in diabetes

Expressed by a pro-inflammatory vascular environment, TF is a main contributor to elevated thrombogenicity in cardiovascular diseases and associated with thromboembolic vascular complications [34, 35]. Notably, patients with poorly controlled diabetes have a high mortality. These individuals show increased TF activity in the blood and circulating TF is associated with disease progression [14, 36, 37].

Zhang et al. first reported the post-transcriptional regulation of TF by miR-19a, a member of the miR-17-92 cluster, in cancer cells [20]. Being investigated as an oncogene at first place, the miR-17-92 cluster has just been brought to the focus of vascular biology. Members appear to be involved in vascular functions, including ischemia responses and angiogenesis [23, 38]. For instance, miR-19b was found to inhibit EC apoptosis [39] and together with miR-20a participates in the control of monocytic TF, which is defective in patients with antiphospholipid syndrome and systemic lupus erythematosus [40]. In the light of previous reports and our data, increased vascular TF expression can no longer be considered a result of induced transcription alone but also a consequence of lacking post-transcriptional control.

Here, we demonstrate that miR-19a reduces expression of TF in endothelial and monocytic cells by binding to the TF transcript in vitro and provide evidence that miR-19a contributes to the control of TF-mediated thrombogenicity in diabetes. Our data suggest that a reduction in endothelial miR-19a leads to increased TF expression and may put patients at risk for thromboembolic events. Accordingly, low circulating miR-19a levels in the blood were found in patients with coronary artery disease or ischemic stroke compared to controls [26, 41]. However, other studies reported that miR-19a and 19b are increased in patients with acute myocardial infarction but suggested a protective role in this setting [42, 43].

### miR-19a and miR-126 are co-expressed in diabetes and have cooperative and differential functions

Vascular inflammation is a hallmark of diabetes and was shown to precede the onset of the disease [44]. Interestingly, we found that miR-19a induced the expression of VCAM1 in endothelial cells. In line, Akhtar et al. reported a hypoxia-induced cell adhesion mediated by miR-19a [45]. Mechanistically, hampering of a (nuclear factor)NFkB negative regulon as employed by miR-19b may be an explanation for our in vitro findings [46]. However, in the patient cohort miR-19a was associated with reduced levels of VCAM. On one hand, miR-19a was shown to also promote anti-inflammatory responses, such as direct targeting of the TNF-α mRNA with subsequent upstream control of the TNFα/NFkB axis [47]. Moreover, down regulation of endothelial TF protein by miR-19a also hampers the pro-inflammatory singling via protease activated receptors [12]. On the other hand, we found miR-126 co expressed with miR-19a in the patients. miR-126 directly targets the VCAM1 mRNA and is even higher expressed in the endothelium than miR-19a what may explain the findings in the our patients [48]. Regulation of miR-19a and miR-126 expression by the glucose-sensitive transcription factor Ets-1 and Ets-2 in ECs could be responsible for the co expression in the patients [49–51].

Finally, miR-19a and miR-126 exhibited a cooperative suppression of the TF transcript in a luciferase reporter assay. In line, deletion of the binding sites for miR-19a/b and miR 20a/b in the TF 3′UTR had a cumulative effect on the reporter activity in RAW 264.7 cells compared to either deletion alone [52]. As shown in Fig. 6b the binding sites of both miRs are closely related to each other. Grimson et al. demonstrated that two different miRs exert a cooperative effect on a transcript given a limited spacing between the two miR bindings sites and suggested cooperative contacts with repressive machinery or removal of occlusive mRNA structures as the underlying mechanism [53]. Our data highlight the cooperative function of miRs to exert (patho)biological functions, including control of thrombogenicity by targeting the TF 3′UTR. In line, Zampetaki et al. found various miRs to be strongly co-expressed in cardiovascular diseases [54]. Alterations in the expression pattern of vascular protective miRs may hence put patients at risk for thromboembolic complications.

## Limitations

In the group with high miR-19a expression, more patients received metformin compared to the group with low miR-19a expression. Metformin was found to reduce pro-inflammatory cascades in vascular cells [55] and decreased TF expression in monocytes in vitro [56]. Vice versa, in the group with high miR-19a expression fewer patients were treated with sulfonylurea. These differences between the two groups may have biased the findings in this study. Moreover, the measurement of TF protein in the plasma using the ELISA from Sekisui Diagnostics may overestimate the TF levels in some settings [57]. However, in addition we used an “in-house” assay to quantify TF activity to overcome these limitations.

## Conclusion

In summary, our study demonstrates that miR-19a decreases procoagulant activity in ECs and monocytes and correlated with reduced TF-dependent thrombogenicity in patients with diabetes. The induction of endothelial VCAM1 in vitro may explain the context-specific role of miR-19a in cardiovascular diseases. However, in the clinical setting of diabetes, co expression of miR-126 with miR-19a leads to control of vascular inflammation and potentiates the post-transcriptional regulation of vascular TF.

## Additional file

**Additional file 1: Figure S1.** Densitometric analysis of the western blot experiments. Density of the bands showing asTF (A) and flTF (B) protein in HMEC transfected with a co miR or miR-19a following stimulation with TNF-α for 6h. Densitometric analysis of asTF (C) and flTF (D) in HMEC transfected with an inhibitor control or anti-miR-19a and stimulation with TNF-α for 6h. (E) VCAM protein in HMEC transfected with miR-19a, miR-126 or both miR-19a and miR-126 together following TNF-α for 6h. Data are represented as mean ± SEM. *p<0.05, **p<0.01, § p<0.05 vs. co miR TNF-α n≥3.
